# Microwave quasi-solid-constructed Ni_2_P–Ni_12_P_5_-supported Os with unique metal–support interaction for anion-exchange membrane seawater electrolysis†

**DOI:** 10.1039/d5sc02930a

**Published:** 2025-06-13

**Authors:** Qing Liu, Xiaowei Fu, Hongdong Li, Jun Xing, Weiping Xiao, Yingxia Zong, Guangying Fu, Jinsong Wang, Qiang Cao, Tianyi Ma, Lei Wang, Zexing Wu

**Affiliations:** a Key Laboratory of Eco-chemical Engineering, Ministry of Education, International Science and Technology Cooperation Base of Eco-chemical Engineering and Green Manufacturing, College of Chemistry and Molecular Engineering, Qingdao University of Science & Technology 53 Zhengzhou Road Qingdao 266042 China splswzx@qust.edu.cn inorchemwl@126.com; b College of Science, Nanjing Forestry University Nanjing 210037 China; c Key Laboratory of Photoelectric Conversion and Utilization of Solar Energy, Qingdao Institute of Bioenergy and Bioprocess Technology, Chinese Academy of Sciences CN-266101 Qingdao China; d Faculty of Materials Science and Engineering, Kunming University of Science and Technology Kunming 650093 China; e School of Mathematics and Physics, Qingdao University of Science & Technology Qingdao 266061 China; f Centre for Atomaterials and Nanomanufacturing (CAN), School of Science, RMIT University Melbourne VIC 3000 Australia tianyi.ma@rmit.edu.au

## Abstract

Highly efficient and corrosion-resistant electrocatalysts for the seawater hydrogen evolution reaction (HER) are crucial for large-scale hydrogen production. Herein, Ni_2_P–Ni_12_P_5_-supported Os (Os/Ni_2_P–Ni_12_P_5_) was synthesized within 30 s *via* an ultrafast and simple microwave quasi-solid approach. This fabricated interface improves the electron transfer efficiency, while metal–support interaction (MSI) between Os and Ni_2_P–Ni_12_P_5_ further optimizes the electronic structure, and then significantly expedites the HER process. The electrocatalyst presents excellent performance in alkaline seawater with a low overpotential of 17 mV to reach the current density of 10 mA cm^−2^. In simulated industrial conditions (1 M KOH + seawater) using an anion exchange membrane water electrolyzer (AEMWE), the constructed Os/Ni_2_P–Ni_12_P_5_ ‖ RuO_2_ cell system required a small voltage of 2.06 V to achieve 1 A cm^−2^. The cost calculation for the produced hydrogen reveals a low price of USD $0.92 per gallon of gasoline equivalent (GGE), which demonstrates its economic advantages for industrialized application. Moreover, various stability measurements revealed that the electrolytic cell system exhibits excellent durability without significant current fluctuations. This corrosion-resistant electrocatalyst with enhanced price activity and mass activity for sustainable seawater electrolysis will pave the way in the design of efficient electrocatalysts with diverse strategies from a novel vision.

## Abstract 2

Ni_2_P–Ni_12_P_5_-supported Os (Os/Ni_2_P–Ni_12_P_5_) as HER catalyst is synthesized within 30 s through an ultrafast and simple microwave quasi-solid approach.

## Graphical abstract

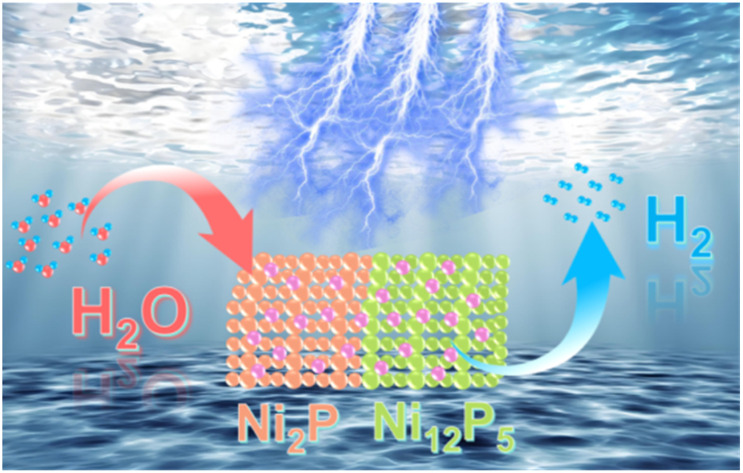

## Introduction

In the context of the escalating demand for energy and the dwindling reserves of carbon-based fuels, contemporary researchers are focusing their efforts on novel and renewable energy sources as alternatives.^1,2^ There is great interest in hydrogen (H_2_) as one such source because it can serve as a substitute for conventional fossil fuels on account of its high energy density.^3^ Among several technologies for obtaining H_2_, electrocatalytic water-splitting (EWS) stands out as one of the most well-established procedures due to its high efficiency and simple process.^4^

Considering the scarcity of freshwater and its low ionic conductivity, researchers have shifted their focus towards seawater electrolysis.^5,6^ However, the complex ionic compositions of seawater have resulted in attendant problems, such as additional side reactions and catalyst poisoning, and these obstacles negatively affect the activity and stability of the electrocatalysts.^7,8^ In view of that, alkaline seawater electrolysis is clearly an effective strategy for mitigating the influence of hard ions such as magnesium ions (Mg^2+)^ and calcium ions (Ca^2+^).^9^

The hydrogen evolution reaction (HER) is an essential step that can significantly affect the entire progress of EWS. Currently, Pt-based catalysts and their compounds remain the benchmark for HER.^10^ However, the scarcity of platinum in reserve and its low resistance to catalyst poisoning pose significant obstacles to the practical application of Pt-based compounds in the HER.^11^ Therefore, the development of non-Pt and highly efficient electrocatalysts for the HER is regarded as a prominent research direction for industrial hydrogen production.

Transition-metal phosphides (TMPs) can serve as electrocatalysts for the HER, and are one of the most promising candidates to replace Pt-based catalysts by virtue of their excellent conductivity and unique physicochemical properties in energy-related applications.^12,13^ However, when TMPs are applied to the HER in electrolytes with complex compositions such as seawater, limited active sites are exposed, and thus, poor catalytic performance is exhibited.^14^ Hence, strategies for adjusting the electronic structure, such as interface engineering,^15^ noble-metal loading,^16^ doping,^17^ and surface vacancy engineering,^18^ are commonly employed to further enhance the electrocatalytic activity.

Among various catalyst modification strategies, noble-metal loading has emerged as a particularly noteworthy approach due to its exceptional efficiency and practical simplicity, which are primarily attributed to the formation of a synergistic metal–support interaction (MSI).^19,20^ Zhang *et al.* successfully synthesized a Fe-doped Co_3_O_4_-supported Ru (Ru/FeCo) catalyst with an effectively modified electronic structure and improved interfacial electron transfer, which were attributed to the MSI between the loaded Ru and FeCo sites, and the catalyst exhibited enhanced activity and stability.^21^ Nevertheless, improvement of performance may not occur if there is a mismatch between the loaded noble metal and the support. There is a long preparation time for reactants and a complex synthesis process for the majority of electrocatalysts, both of which severely restrict their industrial applications. Therefore, simple and feasible approaches are essential to develop and improve the catalytic performance of the TMPs.

In this work, we report a Ni_2_P–Ni_12_P_5_-supported Os (Os/Ni_2_P–Ni_12_P_5_) catalyst synthesized *via* an ultrafast and simple microwave quasi-solid approach for anion-exchange membrane (AEM)-based alkaline seawater electrolysis. Leveraging the cooperation of a heterogeneous interface and noble metal-loading tactic, the fabricated Os/Ni_2_P–Ni_12_P_5_ demonstrates high electron-transfer efficiency with abundant active sites. Moreover, Raman spectroscopy and X-ray photoelectron spectroscopy (XPS) reveal that the MSI is between the Os sites and Ni_2_P–Ni_12_P_5_,^19^ further facilitating electron transfer within the catalyst, and both of which expedite the progress of the HER so that Pt-like catalytic activity is exhibited in alkaline media.

To explore its application prospects in practical production, LSV curves (without iR correction) and corresponding chronoamperometry measurements were obtained under simulated industrial current densities and high temperatures using an anion exchange membrane water electrolyzer (AEMWE). The Os/Ni_2_P–Ni_12_P_5_ ‖ RuO_2_-integrated water electrolysis cell system exhibited enhanced performance and remarkable stability with a low gallon of gasoline equivalent (GGE) price, which adequately demonstrated its economic superiority. This work introduces an ultrafast and simple strategy for the synthesis of electrocatalysts that can be used in the alkaline seawater HER, and offers novel vistas for the design of robust HER catalysts.

## Results and discussion

**Fig. 1:**
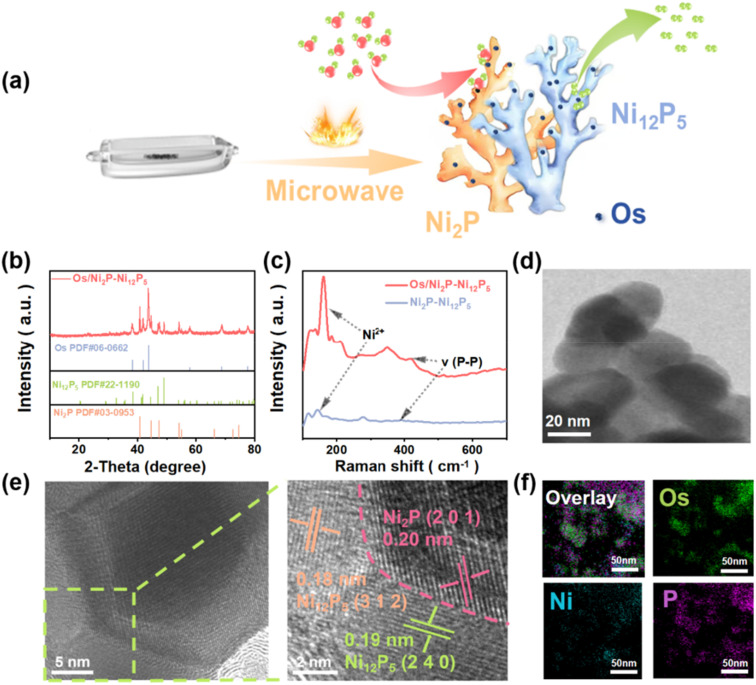
(a) Schematic diagram of the preparation process for Os/Ni_2_P–Ni_12_P_5_. (b) XRD pattern. (c) Raman spectra. (d) TEM image and (e) HRTEM image of Os/Ni_2_P–Ni_12_P_5_. (f) EDS elemental mappings for Os/Ni_2_P–Ni_12_P_5_.

As shown in Fig. 1a, Ni_2_P–Ni_12_P_5_-supported Os (Os/Ni_2_P–Ni_12_P_5_) is synthesized *via* an ultrafast and simple microwave quasi-solid approach. Then, X-ray diffraction (XRD) was employed to determine the phase structures of Os/Ni_2_P–Ni_12_P_5_ and Ni_2_P–Ni_12_P_5_ (Fig. 1b and S1†). As depicted, the substrate predominantly comprises two phases of Ni_2_P (PDF #03-0953) and Ni_12_P_5_ (PDF #22-1190).^22^ Then, diffraction peaks corresponding to osmium (Os) appear (PDF #06-0662) with the original components and remain immutable, indicating that Os was successfully loaded.^23^

Raman spectroscopy was employed to conduct an in-depth investigation into the interactions between Os and Ni_2_P–Ni_12_P_5_. As illustrated in Fig. 1c, wave peaks corresponding to Ni^2+^ at 133 cm^−1^ and P–P at 385 cm^−1^ were detected.^24^ Notably, the loaded Os significantly enhanced the intensity of the detected peaks, and an obvious shift to a high wavenumber occurred, indicating the altered electronic environment and MSI between Os sites and Ni_2_P–Ni_12_P_5_.^25^

Moreover, scanning electron microscopy (SEM), transmission electron microscopy (TEM), and high-resolution TEM (HRTEM) were utilized to analyze the microscopic morphology of Os/Ni_2_P–Ni_12_P_5_ (Fig. 1d, e, and S2†). A dense and compact bulk structure was observed with a distinct interface. The 0.20 nm spacing corresponds to the (201) crystallographic plane of Ni_2_P,^26^ while the 0.18 nm spacing and 0.19 nm spacing were attributed to the (312) and (240) crystallographic planes of Ni_12_P_5_,^22,27^ which further corroborates the XRD results. EDX mapping was also introduced to investigate the elements distributed in Os/Ni_2_P–Ni_12_P_5_ (Fig. 1f), and identified the regular distribution of Ni, P, and Os. Additionally, to determine the weight percentage of Os in Os/Ni_2_P–Ni_12_P_5_, inductively coupled plasma-atomic emission spectroscopy (ICP-AES) was conducted (Table S1). The proportion of elemental Os was determined to be approximately 9.42 wt%.

**Fig. 2:**
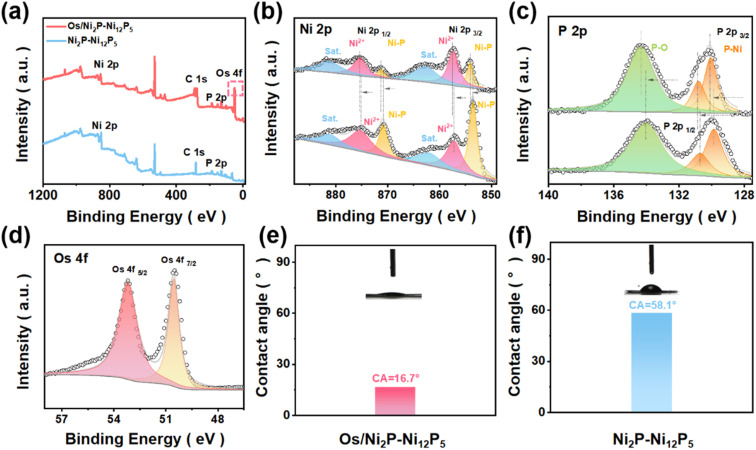
(a) XPS spectra of Os/Ni_2_P–Ni_12_P_5_ and Ni_2_P–Ni_12_P_5_. (b) Ni 2p, (c) P 2p, and (d) Os 4f. The water contact angle measurements of (e) Ni_2_P–Ni_12_P_5_ and (f) Os/Ni_2_P–Ni_12_P_5_.

To conduct a more in-depth investigation into the electronic interactions of Os/Ni_2_P–Ni_12_P_5_ and Ni_2_P–Ni_12_P_5_, XPS tests were performed. Fig. 2a presents a comparison of the two obtained XPS spectra. The characteristic peaks of Os in the spectrum of Os/Ni_2_P–Ni_12_P_5_, and the presence of Ni and P elements were clearly verified. Moreover, the XPS spectra of Ni 2p were compared, and it was deconvoluted into six main peaks (Fig. 2b). The components at the binding energy of 875.5 and 857.2 eV were attributed to the 2p_1/2_ and 2p_3/2_ orbitals of Ni^2+^, while the peaks at 880.2 and 862.2 eV correspond to the satellite peaks, and peaks at 870.9 and 853.6 eV were assigned to Ni–P in Ni_2_P–Ni_12_P_5_.^28,29^ When comparing the Ni 2p spectra of the two catalysts, the loading of Os led to an overall shift towards high binding energy, indicating the interactions between Os and Ni_2_P–Ni_12_P_5_. As depicted in Fig. 2c, the peak at 134 eV was assigned to P–O bonds, and the other two peaks situated near 129.8 and 130.6 eV were attributed to the 2p_3/2_ and 2p_1/2_ of Ni–P.^28^

A similar trend of a shift towards high binding energy was observed in the XPS spectra of P 2p, which further indicates the interactions between Os and Ni_2_P–Ni_12_P_5_. The two peaks at 50.5 and 53.2 eV were attributed to Os 4f_7/2_ and Os 4f_5/2_, demonstrating the existence of Os^(0)^ (Fig. 2d),^30^ which corresponds with the XRD results. Consequently, it was proven that the loading of Os eventually alters the electronic environment, and electrons are transferred from Ni_2_P–Ni_12_P_5_ to the Os sites.^5^ Additionally, MSI exists between Os sites and Ni_2_P–Ni_12_P_5_, which corresponds with the results of Raman spectroscopy.^19^

Furthermore, to investigate the hydrophilicity of the catalysts, water contact angle (CA) tests were carried out. As illustrated in Fig. 2e and f, water droplets were dripped onto the surface of the tablets, and the contact angles measured were 16.7° and 58.1°, respectively. By comparison, it is evident that loaded Os significantly enhances the hydrophilicity of Ni_2_P–Ni_12_P_5_, indicating that the Os/Ni_2_P–Ni_12_P_5_ catalyst exhibited increased electrolyte affinity, and thus accelerated the water-dissociation process.^3^ To further investigate the feasibility of the Os/Ni_2_P–Ni_12_P_5_ catalyst for sustained operation, supplementary tests were carried out after a 10 h HER stability test in 1 M KOH (Fig. S3 and S4†). As shown, the original components and electronic structure of Os/Ni_2_P–Ni_12_P_5_ remained nearly unchanged, demonstrating its possibility for long-term operation.

**Fig. 3:**
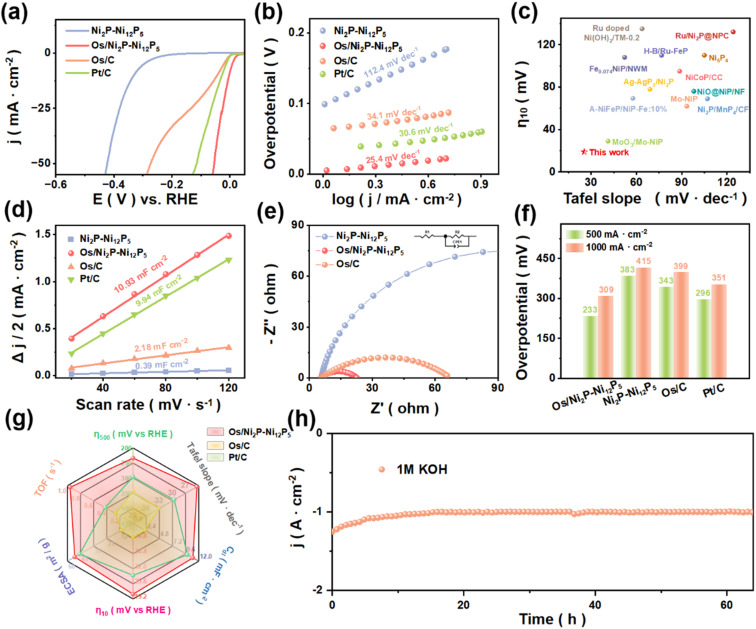
HER performance of catalysts in 1.0 M KOH: (a) LSV curves (95% iR-corrected) of Ni_2_P–Ni_12_P_5_, Os/Ni_2_P–Ni_12_P_5_, Pt/C, and Os/C. (b) Tafel slopes of catalysts. (c) Comparison of the performance of Os/Ni_2_P–Ni_12_P_5_ with recently reported catalysts. (d) *C*_dl_ of catalysts. (e) EIS of catalysts. (f) Performance comparison at 500 and 1000 mA cm^−2^. (g) Comprehensive comparisons of the HER performance of catalysts. (h) Stability test of Os/Ni_2_P–Ni_12_P_5_.

After the loading of Os, the modified Os/Ni_2_P–Ni_12_P_5_ catalyst demonstrated significantly enhanced HER activity compared with Ni_2_P–Ni_12_P_5_, which can be attributed to the MSI between Os and Ni_2_P–Ni_12_P_5_. With a remarkably low overpotential of 19 mV required to achieve a current density of 10 mA cm^−2^, Os/Ni_2_P–Ni_12_P_5_ exhibited a superior electrochemical performance compared with Pt/C (28 mV) and Os/C (48 mV) (Fig. 3a). Then, the Tafel slope was calculated to investigate the reaction kinetics and mechanism (Fig. 3b). A smaller Tafel slope value indicates faster reaction kinetics, and the value of the Tafel slope of Os/Ni_2_P–Ni_12_P_5_ is 25.4 mV dec^−1^, suggesting that it may follow the Volmer-Tafel mechanism.^31^ It is significantly lower than that of Ni_2_P–Ni_12_P_5_ (112.4 mV dec^−1^), demonstrating superior performance and faster reaction kinetics.^32^

In Fig. 3c, different catalysts are compared in terms of their overpotentials at a current density of 10 mA cm^−2^ and the corresponding Tafel slopes. The Os/Ni_2_P–Ni_12_P_5_ catalyst outperformed most of the reported catalysts, demonstrating its satisfactory electrochemical performance for HER. Furthermore, cyclic voltammetry (CV) tests were carried out with scan rates varying from 20 to 120 mV s^−1^ (Fig. S5†). Upon calculation, the related electrochemical double-layer capacitance (*C*_dl_) and electrochemically active surface area (ECSA) of Os/Ni_2_P–Ni_12_P_5_ were obtained (Fig. 3d and S6†). The *C*_dl_ of Os/Ni_2_P–Ni_12_P_5_ was determined to be 10.93 mF cm^−2^, which is much higher than that of Ni_2_P–Ni_12_P_5_, indicating that Os/Ni_2_P–Ni_12_P_5_ exposes more active sites, thereby enhancing the electrocatalytic activity.^33^

Subsequently, electrochemical impedance spectroscopy (EIS) was employed to measure the charge transfer resistance (*R*_ct_) value (Fig. 3e). The Os/Ni_2_P–Ni_12_P_5_ exhibited a low *R*_ct_ value in the low-frequency range relative to the contrast catalysts, which demonstrates its rapid electron transfer rate in alkaline media. As depicted in Fig. 3f and S7,† Os/Ni_2_P–Ni_12_P_5_ required overpotentials of only 233 mV and 309 mV to reach current densities of 0.5 A cm^−2^ and 1.0 A cm^−2^, respectively, indicating its satisfactory electrocatalytic activity under industrial current density. In Fig. 3g, the electrochemical performances of all the studied catalysts are comprehensively presented. Notably, the highest turnover frequency (TOF) value among the catalysts measured above was obtained for Os/Ni_2_P–Ni_12_P_5_, illustrating that Os/Ni_2_P–Ni_12_P_5_ possesses a significantly high rate of converting reactants to products per active site per unit of time.^5^ Under the fixed voltages of −1.20 V and −1.05 V, the current density fluctuations at 0.5 A cm^−2^ and 10 mA cm^−2^ of Os/Ni_2_P–Ni_12_P_5_ are nearly negligible (Fig. 3h and S8†), proving its excellent stability for the HER.

**Fig. 4:**
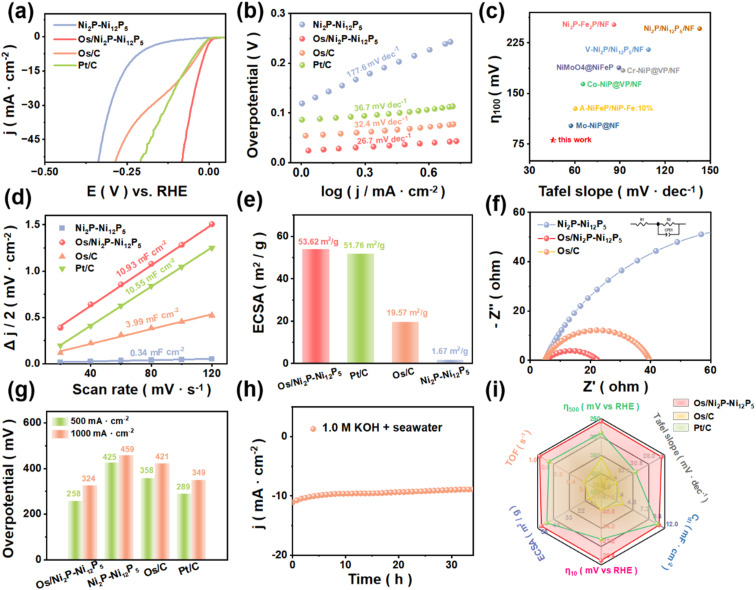
HER performance of catalysts in 1.0 M KOH + seawater: (a) LSV curves (95% iR-corrected) of catalysts. (b) Tafel slope of catalysts. (c) Comparison of Os/Ni_2_P–Ni_12_P_5_ performance with recently reported catalysts. (d) *C*_dl_ of catalysts. (e) EIS of catalysts. (f) ECSA of catalysts. (g) Performance comparison at 500 and 1000 mA cm^−2^. (h) Stability test of Os/Ni_2_P–Ni_12_P_5_. (i) Comprehensive comparisons of the HER performance of catalysts.

Inspired by the excellent performance in alkaline freshwater, further explorations in alkaline seawater under the same parameters were conducted. As depicted in Fig. 4a, Os/Ni_2_P–Ni_12_P_5_ demonstrated a superior electrochemical performance and exhibited significantly enhanced HER kinetics compared with Ni_2_P–Ni_12_P_5_. It exhibited an extremely low overpotential of only 17 mV @ 10 mA cm^−2^. The overpotentials required for Ni_2_P–Ni_12_P_5_, Pt/C, and Os/C are 233 mV, 50 mV, and 44 mV, respectively. Moreover, the Tafel slope was calculated (Fig. 4b). The value of Os/Ni_2_P–Ni_12_P_5_ is 26.7 mV dec^−1^, suggesting that it may follow the Volmer-Tafel mechanism in 1 M KOH + seawater, which has fastest HER kinetics among Ni_2_P–Ni_12_P_5_ (177.6 mV dec^−1^), Os/C (32.4 mV dec^−1^), and Pt/C (36.7 mV dec^−1^).^32,34^

As depicted in Fig. 4c, Os/Ni_2_P–Ni_12_P_5_ distinctly rises above all other values, with the smallest overpotentials at a current density of 100 mA cm^−2^ and corresponding Tafel slopes when compared with the newly reported electrocatalysts for alkaline seawater electrolysis. Analogously, CV was carried out (Fig. S9†). Upon calculation, the *C*_dl_ (Fig. 4d) of Os/Ni_2_P–Ni_12_P_5_ is 10.93 mF cm^−2^, proving that additional active sites are exposed. Furthermore, the ECSAs (eqn (S1)) of various catalysts were estimated by the calculated *C*_dl_. As shown in Fig. 4e, Os/Ni_2_P–Ni_12_P_5_ achieved the highest ECSA value of 53.62 m^2^ g^−1^, followed by Pt/C (51.76 m^2^ g^−1^), and then Os/C (19.57 m^2^ g^−1^), with Ni_2_P–Ni_12_P_5_ remaining last (1.67 m^2^ g^−1^), further illustrating that a greater amount of active sites are exposed with Os/Ni_2_P–Ni_12_P_5_. The EIS tests also corresponded with the regularity of the mentioned measurements (Fig. 4f). Benefiting from MSI, the Os/Ni_2_P–Ni_12_P_5_ catalyst exhibits a far lower *R*_ct_ value than that of Ni_2_P–Ni_12_P_5_, which demonstrates its improved electron transfer efficiency. At industrial current densities of 0.5 A cm^−2^ and 1.0 A cm^−2^ (Fig. 4g and S10†), Os/Ni_2_P–Ni_12_P_5_ requires overpotentials of only 258 mV and 324 mV, respectively, illustrating its promising prospects at industrial current density.

As depicted in Fig. 4h, the Os/Ni_2_P–Ni_12_P_5_ catalyst shows excellent stability, with negligible current attenuation under continuous operation for more than 30 h under a fixed voltage of −1.07 V. Then, six main aspects of electrocatalytic activity of the different catalysts were comprehensively compared (Fig. 4i), demonstrating that the Os/Ni_2_P–Ni_12_P_5_ catalyst exhibits a prominent performance. Moreover, supplementary tests in acidic (Fig. S11–19†) and neutral (Fig. S20–25†) environments were conducted to further explore the application potential of Os/Ni_2_P–Ni_12_P_5_, which requires low overpotentials of 65 mV in 0.5 M H_2_SO_4_ and 136 mV in 1.0 M PBS to reach 10 mA cm^−2^. The results obtained proved that Os/Ni_2_P–Ni_12_P_5_ is capable of catalyzing the desired reaction over a wide pH fluctuation, indicating its promising prospects for applications in complex environments.

**Fig. 5:**
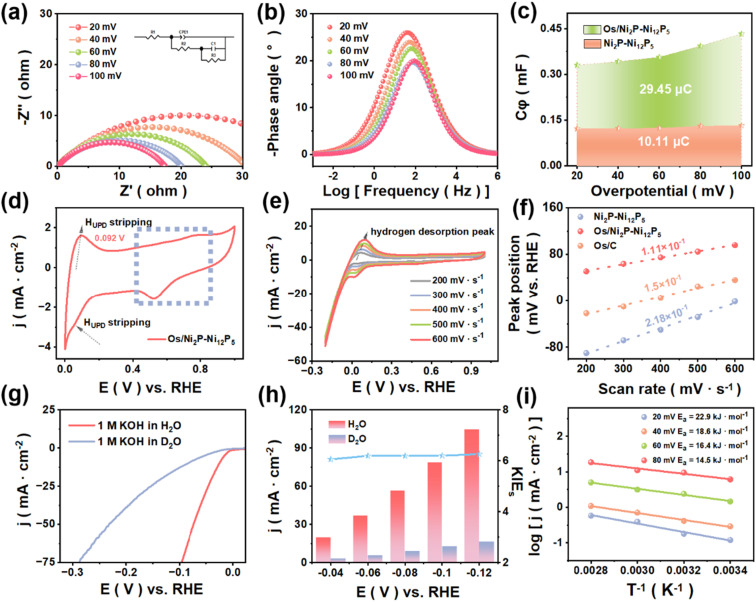
(a) *In situ* EIS of Os/Ni_2_P–Ni_12_P_5_ in 1.0 M KOH. (b) Bode plots of Os/Ni_2_P–Ni_12_P_5_. (c) Plots of *C*_φ_*vs.* overpotential of Os/Ni_2_P–Ni_12_P_5_ and Ni_2_P–Ni_12_P_5_ during the HER in 1.0 M KOH. (d) CV curves at a scan rate of 50 mV s^−1^. (e) CV curves recorded at various scan rates under a saturated Ar atmosphere, and (f) fitted plots of H desorption peak positions at different scan rates of catalysts. (g) LSV curves (95% iR-corrected) of Os/Ni_2_P–Ni_12_P_5_ measured in 1.0 M KOH + H_2_O and 1.0 M KOH + D_2_O. (h) Calculated KIE values under the corresponding potentials of Os/Ni_2_P–Ni_12_P_5_. (i) Arrhenius plots of Os/Ni_2_P–Ni_12_P_5_.

To investigate the behavior of H* during the HER, *in situ* EIS tests were conducted, and an equivalent circuit model was established to simulate the Nyquist plots of Os/Ni_2_P–Ni_12_P_5_ and Ni_2_P–Ni_12_P_5_ (Fig. 5a, S26, and S27†). As depicted, the impedance of Os/Ni_2_P–Ni_12_P_5_ exhibited a decreasing trend as the potential increased, and it is far lower than that of Ni_2_P–Ni_12_P_5_. This emphatically demonstrates that the MSI between Os and Ni_2_P–Ni_12_P_5_ significantly optimizes the electron transfer process.^19^ Similarly, as shown in Fig. 5b and S28,† the phase peak angle in the Bode plots also shows the same trend, decreasing with the increase in voltage, which indicates a reduction in the electron transfer impedance.^35^

Furthermore, the charge transfer kinetics of Os/Ni_2_P–Ni_12_P_5_ was investigated. Specifically, the adsorption behavior of hydrogen intermediates on active sites can be reflected by fitting the *R*_ct_ and hydrogen adsorption pseudocapacitance (*C*_φ_). The hydrogen adsorption charge (*Q*_H_), calculated by integrating *C*_φ_, is a parameter used to quantitatively describe the amount of H species absorbed on the catalyst surface during the HER.^36^ As exhibited in Fig. 5c, the *Q*_H_ value of Os/Ni_2_P–Ni_12_P_5_ is approximately 2.9 times higher than that of Ni_2_P–Ni_12_P_5_, verifying the enhanced H* coverage at identical overpotentials, and thus confirming the significantly increased hydrogen adsorption.^37^

Then, CV curves at 50 mV s^−1^ were obtained (Fig. 5d, S29, and S30†). Os/Ni_2_P–Ni_12_P_5_ exhibited obvious hydrogen underpotential deposition (H_UPD_) peaks, while no peak was observed for Ni_2_P–Ni_12_P_5_, indicating the promoted H* generation by the loading of the Os HER process, which corresponds with the plots of *C*_φ_. Its hydrogen desorption peak negatively shifted to a potential lower than that of Os/C, representing a lessened hydrogen binding energy (HBE), which accelerated the HER process.^38^ Furthermore, ranges of scanning rates were sampled for different catalysts in Ar-saturated 1.0 M KOH (Fig. 5e, S31, and S32†). A clear peak can be observed, and the position of the peak shifted to the high-voltage direction as the sweep speed increased, while the hydrogen desorption peak was absent for bare Ni_2_P–Ni_12_P_5_, suggesting that there is a higher degree of hydrogen spillover for Os/Ni_2_P–Ni_12_P_5_.^39^

As shown in Fig. 5f, the peak positions at ranges of scan rates were compared, and the curve-fitting slopes were adopted to assess the hydrogen desorption kinetics. There was a significantly reduced slope for Os/Ni_2_P–Ni_12_P_5_ among the different catalysts, indicating its accelerated kinetics.^39^ Moreover, the LSV curves (95% iR-corrected) of catalysts in 1.0 M KOH–H_2_O and 1.0 M KOH–D_2_O were obtained (Fig. 5g, S33, and S35†), which are known as kinetic isotope effects (KIE). The current density at several potentials in the two electrolytes and corresponding KIE values (*J*_H_2_O_/*J*_D_2_O_) were clearly compared (Fig. 5h, S34, and S36†).

Interestingly, the same conclusion can be drawn regarding all of the measured points, which is that the current densities in the 1.0 M KOH + D_2_O solution are notably smaller for each catalyst at the same potentials, with all of the values surpassing 1, indicating that the H* transfer process is the rate-determining step (RDS) of the HER process. When compared with Os/C and Ni_2_P–Ni_12_P_5_, the KIE values of Os/Ni_2_P–Ni_12_P_5_ proved to be the largest, illustrating that the HER kinetics of Os/Ni_2_P–Ni_12_P_5_ is vulnerable by the H* transfer process.^40^

In addition, the catalytic performances of Os/Ni_2_P–Ni_12_P_5_ and Ni_2_P–Ni_12_P_5_ at a temperature gradient were compared to explore the effects of loaded Os on the activation energy (*E*_a_) of the HER (Fig. 5i), both of which follow a decreasing trend as the temperature rises. By applying the Arrhenius equation (eqn (S2)), the *E*_a_ values at different added potentials within the selected temperature ranges were calculated. Notably, the values of Os/Ni_2_P–Ni_12_P_5_ at any added potentials are far smaller than those of Ni_2_P–Ni_12_P_5_ (Fig. S37†), demonstrating that loaded Os sharply reduced the *E*_a_ and thereby decreased the energy barrier of the desired reaction.^41^

**Fig. 6:**
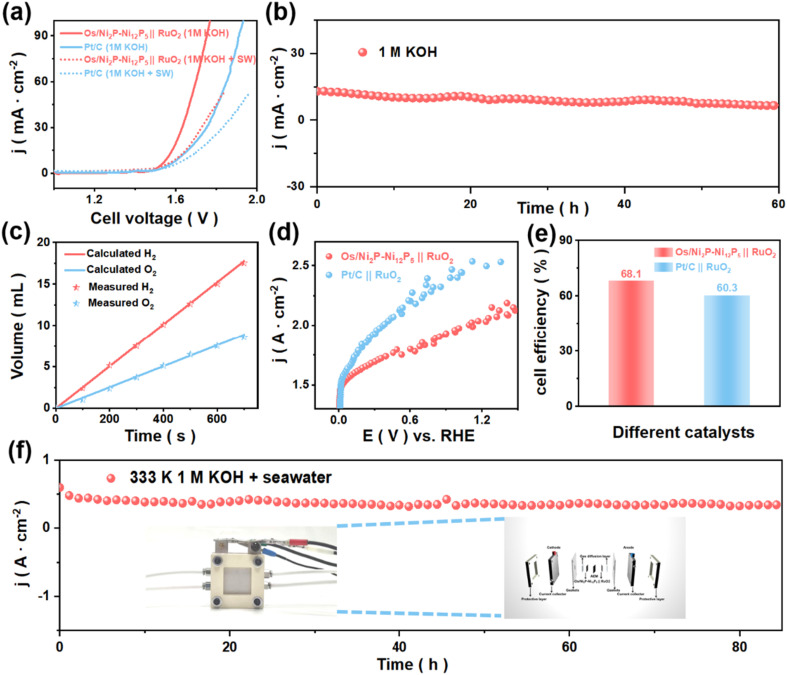
(a) LSV curves (95% iR-corrected) of Os/Ni_2_P–Ni_12_P_5_ ‖ RuO_2_ and Pt/C ‖ RuO_2_ for overall water-splitting in 1.0 M KOH and in 1.0 M KOH + seawater. (b) Stability test of Os/Ni_2_P–Ni_12_P_5_ ‖ RuO_2_. (c) Theoretical and actual values of the volume of gas as a function of time. (d) LSV curves (without iR correction) of overall seawater electrolysis (1 M KOH + seawater) for Os/Ni_2_P–Ni_12_P_5_ ‖ RuO_2_ and Pt/C ‖ RuO_2_ in an AEMWE. (e) Comparison of cell catalytic efficiency between Os/Ni_2_P–Ni_12_P_5_ ‖ RuO_2_ and Pt/C ‖ RuO_2_. (f) Stability test of AEMWE-based alkaline seawater electrolysis at 500 mA cm^−2^. The inset shows a schematic diagram of an alkaline AEMWE.

Enlightened by the excellent HER performance of Os/Ni_2_P–Ni_12_P_5_ in alkaline media, further explorations of its industrial application were carried out, and are presented in Fig. 6. By applying Os/Ni_2_P–Ni_12_P_5_ as the cathode and RuO_2_ as the anode, an Os/Ni_2_P–Ni_12_P_5_ ‖ RuO_2_ integrated system for overall water electrolysis was established.^42^Fig. 6a clearly shows the electrochemical performance of Os/Ni_2_P–Ni_12_P_5_ ‖ RuO_2_. It required relatively low voltages of 1.55 V and 1.60 V to reach 10 mA cm^−2^ in 1 M KOH and 1 M KOH + seawater, respectively, while the Pt/C ‖ RuO_2_ system required 1.61 V and 1.65 V for the same current density in the corresponding electrolytes. Subsequently, a relative stability test was conducted. As shown in Fig. 6b, the Os/Ni_2_P–Ni_12_P_5_ ‖ RuO_2_ cell system exhibited a negligible decrease in current density under a constant voltage of 1.63 V during continuous operation for over 60 h, which clearly reveals its excellent electrochemical stability.

Moreover, to measure the Faraday efficiency of the Os/Ni_2_P–Ni_12_P_5_ ‖ RuO_2_ electrolytic cell system, the water drainage method was employed to collect the amounts of generated H_2_ and O_2_ (Fig. 6c and S38†). The theoretical and measured values of hydrogen and oxygen generation nearly overlap, indicating that the Faraday efficiency is close to 100%. Additionally, the electrolytic cell system can be powered by other forms of energy (Fig. S39†). Simulated wind, thermal, and solar energies were used to generate H_2_ by the Os/Ni_2_P–Ni_12_P_5_ ‖ RuO_2_ electrolytic cell system, demonstrating its potential for practical applications.^43–46^

To explore its potential for industrial applications, LSV curves (without iR correction) of overall seawater electrolysis were measured under simulated industrial conditions using the AEMWE.^47^ Compared with Pt/C ‖ RuO_2_, the electrolysis system assembled with Os/Ni_2_P–Ni_12_P_5_ and RuO_2_ can be driven at a lower potential of 2.06 V to reach the same current density (Fig. 6d), exhibiting a relatively high cell efficiency of 68.1% (Fig. 6e). It was calculated that the price per GGE of the H_2_ produced by Os/Ni_2_P–Ni_12_P_5_ is USD $0.92, which is much lower than the 2026 target of USD $2.0/GGE set by the U.S. Department of Energy (DOE).^48^

Additionally, to deeply probe into analyzing the economic efficiency, the mass activity and price activity of Os/Ni_2_P–Ni_12_P_5_ were calculated (Fig. S40†). When the selected voltage was 2.0 V, the Os/Ni_2_P–Ni_12_P_5_ ‖ RuO_2_ electrolytic cell system (3.01 A mg^−1^ and 143.3 A dollar^−1^) achieved a much higher mass activity and price activity than that of Pt/C ‖ RuO_2_ (0.92 A mg^−1^ and 9.9 A dollar^−1^), indicating its high catalytic activity and cost-effectiveness.^49^

Apart from that, stability is also an important parameter that plays a key role in practical applications. Therefore, a stability test at 1.86 V of AEMWE-based alkaline seawater electrolysis at 500 mA cm^−2^ was carried out. As shown, the assembled AEMWE system exhibited satisfactory long-term stability for over 80 h under simulated conditions (60 °C, 1 M KOH + seawater) with a negligible decrease in current density (Fig. 6f), which adequately demonstrates its potential for industrialized application.

## Conclusions

In this work, we propose an innovative approach for constructing Ni_2_P–Ni_12_P_5_-supported Os (Os/Ni_2_P–Ni_12_P_5_) *via* a microwave-assisted method utilizing the effects of a heterogeneous interface and a noble metal loading strategy. This fabricated interface exhibits MSI between Os sites and Ni_2_P–Ni_12_P_5_, which results in a significantly enhanced HER performance when compared to Ni_2_P–Ni_12_P_5_. Only low overpotentials of 19 mV, 17 mV, and 65 mV in 1 M KOH, 1 M KOH + seawater, and 0.5 M H_2_SO_4_, respectively, are required to reach 10 mA cm^−2^. Compared with Pt/C ‖ RuO_2_, the electrolysis cell system assembled with Os/Ni_2_P–Ni_12_P_5_ and RuO_2_ can be driven at a lower potential of 2.06 V to reach the same current density, exhibiting a relatively high cell efficiency of 68.1%. The cost calculation for the produced hydrogen reveals a low price of USD $0.92 per GGE, which demonstrates its promising prospects for industrialized application. Moreover, it also exhibits excellent stability in various electrolytes. This work paves a pathway for the design of efficient electrocatalysts with enhanced performance by strengthening the metal-substrate interaction for H_2_ generation from copious amounts of seawater.

## Author contributions

Q. Liu performed the investigation and material synthesis, carried out electrochemical tests, analyzed the data, and wrote the original draft. X. Fu completed data collation, conceptualization, and validation. H. Li, J. Xing, W. Xiao, Y. Zong, G. Fu, J. Wang, Q. Cao, and T. Ma provided valuable guidance and writing assistance. Z. Wu performed the conceptualization, writing – review and editing, supervision, and funding. L. Wang provided writing – review and editing, funding acquisition, and supervision. All authors discussed the results and provided comments on the manuscript.

## Conflicts of interest

The authors declare no conflicts of interest.

## Supplementary Material

SC-OLF-D5SC02930A-s001

## Data Availability

The data supporting this article have been included as part of the ESI.†
