# Supplementary material for: Microwave quasi-solid-constructed Ni2P–Ni12P5-supported Os with unique metal–support interaction for anion-exchange membrane seawater electrolysis
Source: Chem Sci. 2025 Jun 13;16(29):13306–15. doi: 10.1039/d5sc02930a (PMC12186116; doi:10.1039/d5sc02930a)
Supplement: SC-016-D5SC02930A-s001 [file SC-016-D5SC02930A-s001.pdf]

Supplementary Information (SI) for Chemical Science.
This journal is © The Royal Society of Chemistry 2025

Supporting Information

Microwave Quasi-solid Constructed Ni_2P-$Ni_{12}P_5$ Supported Os with Unique Metal-Support Interaction for Anion-exchange Membrane Seawater Electrolysis

Qing Liu[a, 1], Xiaowei Fu[a, 1], Hongdong Li[a], Jun Xing[a], Weiping Xiao[b], Yingxia Zong[a], Guangying Fu[c], Jinsong Wang[d], Qiang Cao[e], Tianyi Ma[f, *], Lei Wang[a, *], Zexing Wu[a, *]

a. Key Laboratory of Eco-chemical Engineering, Ministry of Education, International Science and Technology Cooperation Base of Eco-chemical Engineering and Green Manufacturing, College of Chemistry and Molecular Engineering, Qingdao University of Science & Technology 53 Zhengzhou Road, 266042, Qingdao, P. R. China E-mail: splswzx@qust.edu.cn; inorchemwl@126.com.

b. College of Science, Nanjing Forestry University, Nanjing, 210037, P. R. China.

c. Key Laboratory of Photoelectric Conversion and Utilization of Solar Energy, Qingdao Institute of Bioenergy and Bioprocess Technology, Chinese Academy of Sciences, CN-266101 Qingdao, China.

d. Faculty of Materials Science and Engineering, Kunming University of Science and Technology, Kunming 650093, PR China

e. School of Mathematics and Physics, Qingdao University of Science & Technology, Qingdao 266061, China

f. Centre for Atomaterials and Nanomanufacturing (CAN), School of Science, RMIT University, Melbourne, VIC 3000, Australia E-mail: tianyi.ma@rmit.edu.au

1. The authors contributes equally to this work.

*Corresponding authors

Experimental

Physical Characterization

X-ray diffraction (XRD) analysis (Rigaku SmartLab SE, Japan) was employed to investigate the crystal structure of the synthesized sample. Scanning electron microscopy (SEM) (TESCAN MIRA LMS, Czech) was utilized to characterize the morphology of the prepared sample. Morphology and Energy Dispersive X-ray Spectroscopy (EDS) analyses of the prepared sample were performed using transmission electron microscopy (TEM) (FEI Talos F200X G2, USA). X-ray photoelectron spectroscopy (XPS) analysis (Thermo Scientific K-Alpha, USA) was conducted to investigate the surface elemental state of the sample. Raman spectroscopy (LabRam HR Evolution) was employed to analyze the vibrational and rotational states between molecules of the sample. The content of elements leached in samples and electrolytes was obtained by inductively coupled plasma atomic emission spectrometry (ICP-MS, Aglient7850).

Materials and medicines

Nickle chloride hexahydrate ($NiCl_2·6H_2O$, AR 99%) and platinum-carbon (Pt/C, 20%) were purchased from Macklin, while sodium hypophosphite monohydrate ($NaH_2PO_2·H_2O$, 99%) and potassium osmate (VI) dihydrate ($K_2OsO_4·2H_2O$, ⩾99%) were purchased from Aladdin. Ruthenium (IV) oxide (RuO_2, 99.9% trace metals basis) was purchased from SIGMA-ALDRICH. The NiFe foam was purchased from Suzhou Keshenghe Metal Materials (Ni:Fe=7:3).

Preparation of Ni_2P-$Ni_{12}P_5$

23.7 mg $NiCl_2·6H_2O$ and 106 mg $NaH_2PO_2·H_2O$ are grounded in a mortar. Then, 40 μL deionized water is added to the mixture and heated in a 700 W media microwave oven for almost 30 s. The reacted mixture is washed and stirred in deionized water for 30 min and then filtered, then dried in a vacuum oven at 60 °C.

The related parameters of media microwave oven lie follows:

Model: M1-L213C

Rated Voltage/Frequency: 220V~50Hz

Microwave Operating Frequency: 2450MHz

Rated Input Power: 1150W

Microwave Output Power: 700W

Preparation of Os/Ni_2P-$Ni_{12}P_5$

23.7 mg $NiCl_2{\cdot}6H_2O$, 106 mg $NaH_2PO_2{\cdot}H_2O$ and 10 mg $K_2OsO_4{\cdot}2H_2O$ are grounded in a mortar. Then, 40 μL deionized water is added to the mixture and heated in a daily used media microwave oven for almost 30 s. The reacted mixture is washed and stirred in deionized water for 30 min and then filtered, then dry in a vacuum oven at 60 °C.

Electrocatalytic Measurements

5 mg catalyst and 1 mg Vulcan XC-72R carbon black are dispersed in 20 μL Nafion solution and isopropanol solution (1 mL), and then sonicate for 30 minutes to form a uniform ink. Then, 20 μL ink is dropped onto a glassy carbon electrode (containing 0.05 mg of catalyst).

Electrochemical experiments are conducted using an electrochemical workstation (CHI 760E) with a standard three-electrode system to study the electrocatalytic performances of HER. Linear sweeping voltammogram (LSV) curves are recorded with a scan rate of 5 mV s^{-1} with 95% iR-corrected. Electrochemical impedance spectroscopy (EIS) measurements are carried out in frequency ranges of from 100 kHz to 0.01 Hz with an amplitude of 5 mV.

The high current density tests are measured in Gamry 3000 with Ag/AgCl electrode as reference electrode, (E (*vs*. RHE) = E (*vs*. Hg/HgO) + 0.098 V + 0.0591*pH) and carbon paper used as a substrate to load the catalysts. The size of the carbon paper is 1*1 cm and contained 2 mg catalysts. Commercial Pt/C and Os/C are tested by the same way.

The anion-exchange membrane (AEM, Sustainion X37-50) water electrolyzer system is consisted of cathode (Os/Ni_2P-$Ni_{12}P_5$ on NiFe Foam 1 mg cm^{-2}) / (Pt/C on NiFe Foam, 1 mg cm^{-2}) and anode (RuO_2 on NiFe Foam, 1 mg cm^{-2}). The electrolyte (1.0 M KOH + seawater) is supplied under 60 °C.

Calculation of electrochemically active surface area (ECSA)

Equation 1: ECSA= $C_{dl} \times S / C_s$

S is area of working electrode (0.19625 cm^2). And C_s is the constant (1.0 M KOH) that converts the capacitance to ECSA with a value of 0.004 mF cm^{-2}.[1]

Calculation of electrochemical activation energies (E_a)

Equation 2: $E_a = -2.303R \left[\frac{\partial \log(j)}{\partial(\frac{1}{T})} \right]$

R: the molar gas constant (8.314 J mol K^{-1}); j: the current density (mA cm^{-2}); T: the temperature of the reaction (K).[2]

Calculation of TOF

Total hydrogen turnover[3]

$$= \left(|j| \frac{mA}{cm2}\right) \left(\frac{\frac{1\ C}{s}}{1000\ mA}\right) \left(\frac{1\ mol\ e-}{96485.3\ C}\right) \left(\frac{1\ mol}{2\ mol\ e-}\right) \left(\frac{6.022 \times 1023\ molecules\ H2}{1\ mol\ H2}\right)$$

$$= 3.12 \times 10^{15} \frac{H2/s}{cm2} \text{ per } \frac{mA}{cm2}$$

Active sites ($Os/Ni_2P\text{-}Ni_{12}P_5$)

$$= \left(\frac{\text{catalyst loading per geometric area} \times \left(\frac{g}{cm2}\right) \times \text{Os wt\%}}{\text{Os Mw} \left(\frac{g}{mol}\right)}\right) \left(\frac{6.022 \times 1023\ \text{Os atoms}}{1\ \text{mol Os}}\right)$$

=

$$\left(\frac{0.509 \times \left(\frac{10 - 3g}{cm2}\right) \times 9.42\ wt\%}{190.2}\right)\left(\frac{6.022 \times 1023\ Os\ atoms}{1\ mol\ Os}\right)$$

$= 1.52\times10^{17}$ Os sites per cm^2

TOF ($Os/Ni_2P\text{-}Ni_{12}P_5$) = $\left(\frac{3.12 \times 1015}{1.52 \times 1017}\right) \times|j|$ = $\mathbf{0.0205\times|j|}$

$Os/Ni_2P\text{-}Ni_{12}P_5$ (active sites) = 1.52×10^{17} Os sites per cm^2

Os/C (active sites) = 4.83×10^{17} Os sites per cm^2

Pt/C (active sites) = 3.14×10^{17} Pt sites per cm^2

Calculation of AEM electrolyzer efficiency and H_2 cost.

Electrolyzer efficiency [4]

$Os/Ni_2P\text{-}Ni_{12}P_5$ || RuO_2

H_2 production rate @ 0.5 A cm^{-2}

= (j A cm^{-2}) (1 e^-/1.602 $\times10^{-19}$ C) (1 H_2/2 e^-)

= 0.5 A cm^{-2} / (1.602 $\times$ 10^{-19} C x 2)

= 2.59 $\times$ 10^{-6} mol H_2 cm^{-2} s^{-1}

Lower Heating Value (LHV) of H_2 = 120 kJ g^{-1} H_2

= 2.42 $\times$ 10^5 J mol^{-1} H_2

H_2 power out = (2.59 $\times$ 10^{-6} mol cm^{-2} s^{-1}) $\times$ (2.42 $\times$ 10^5 J mol^{-1}) = 0.627 W cm^{-2}

Electrolyzer Power of $Os\text{-}OsP_2$ || RuO_2

Electrolyzer Power ($Os\text{-}OsP_2$ || RuO_2) @ 0.5 A cm^{-2}

= (0.5 A cm^{-2}) (1.84 V)

= 0.92 W cm^{-2}

Efficiency of $Os\text{-}OsP_2$ || RuO_2

= (H_2 Power Out) / (Electrolyzer Power)

= 0.627 W cm^{-2}/0.92 W cm^{-2}

= 68.1%

Price per gasoline-gallon equivalent (GGE) H_2

= 1GGE H_2 / H_2 production rate × Electrolyzer power × Electricity bill

= 0.997 kg / (2.59 × 10^{-6} mol H_2.cm^{-2} s^{-1} × 2 kg/mol) ×0.92 W cm^{-2} × \$ 0.02 / kW h

= \$ 0.97 /GGE H_2<< \$ 2 /GGE H_2 of U.S. Department of Energy (DOE) by 2026

Pt/C || RuO_2

H_2 production rate @ 0.5 A cm^{-2}

= (j A cm^{-2}) (1 e^-/1.602 ×10^{-19} C)(1 H_2/2 e^-)

= 0.5 A cm^{-2} / (1.602 × 10^{-19} C x 2)

= 2.59 × 10^{-6} mol H_2 cm^{-2} s^{-1}

LHV of H_2 = 120 kJ g^{-1} H_2

= 2.42 × 10^5 J mol^{-1} H_2

H_2 power out = (2.59 × 10^{-6} mol cm^{-2} s^{-1}) × (2.42 × 10^5 J mol-1) = 0.627 W cm^{-2}

Electrolyzer Power of Pt/C || RuO_2

Electrolyzer Power (Pt/C || RuO_2) @ 0.5 A cm^{-2}

= (0.5 A cm^{-2}) (2.08 V)

= 1.04 W cm^{-2}

Efficiency of Pt/C || RuO_2

= (H_2 Power Out) / (Electrolyzer Power)

= 0.627 W cm^{-2}/1.04 W cm^{-2}

= 60.28%

Price per gasoline-gallon equivalent (GGE) H_2

= 1GGE H_2 / H_2 production rate × Electrolyzer power × Electricity bill

= 0.997 kg / (2.59 × 10^{-6} mol H_2.cm^{-2} s^{-1} × 2 kg/mol) × 0.905 W cm^{-2} × \$ 0.02 / kW h

= \$ 1.097 /GGE H_2

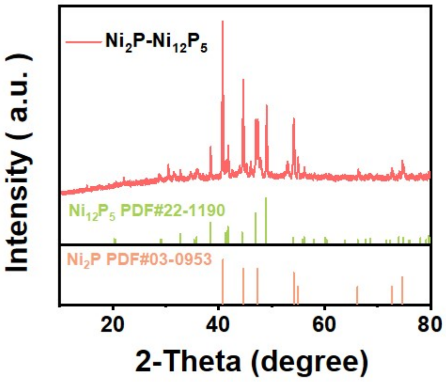

Figure S1 XRD pattern of Ni_2P-$Ni_{12}P_5$.

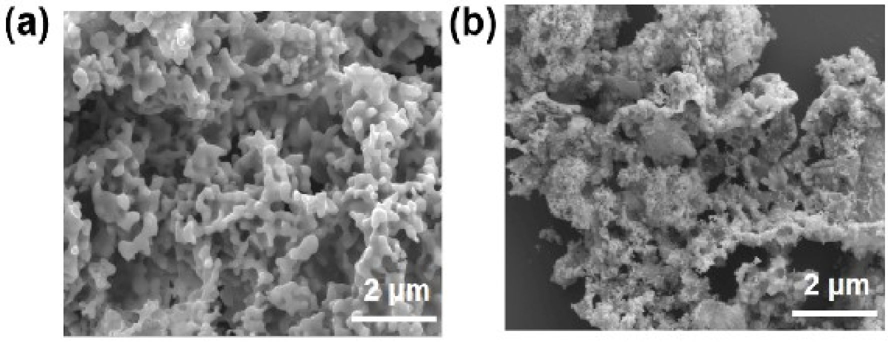

Figure S2 SEM images of (a) Ni_2P-$Ni_{12}P_5$ and (b) Os/Ni_2P-$Ni_{12}P_5$.

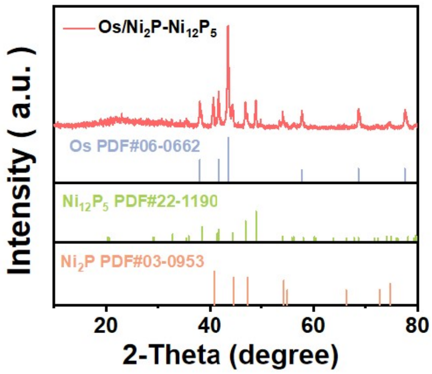

Figure S3 XRD pattern of Os/Ni_2P-$Ni_{12}P_5$ after 10 h stability test (the fixed voltage is -1.05 V) in 1 M KOH.

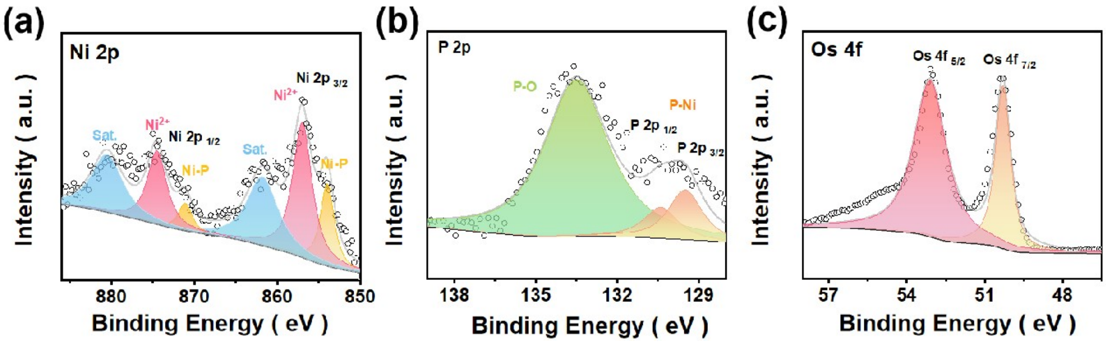

Figure S4 XPS spectra of $Os/Ni_2P-Ni_{12}P_5$ (a) Ni 2p (b) P 2p , (c) Os 4f after 10 h stability test in 1 M KOH.

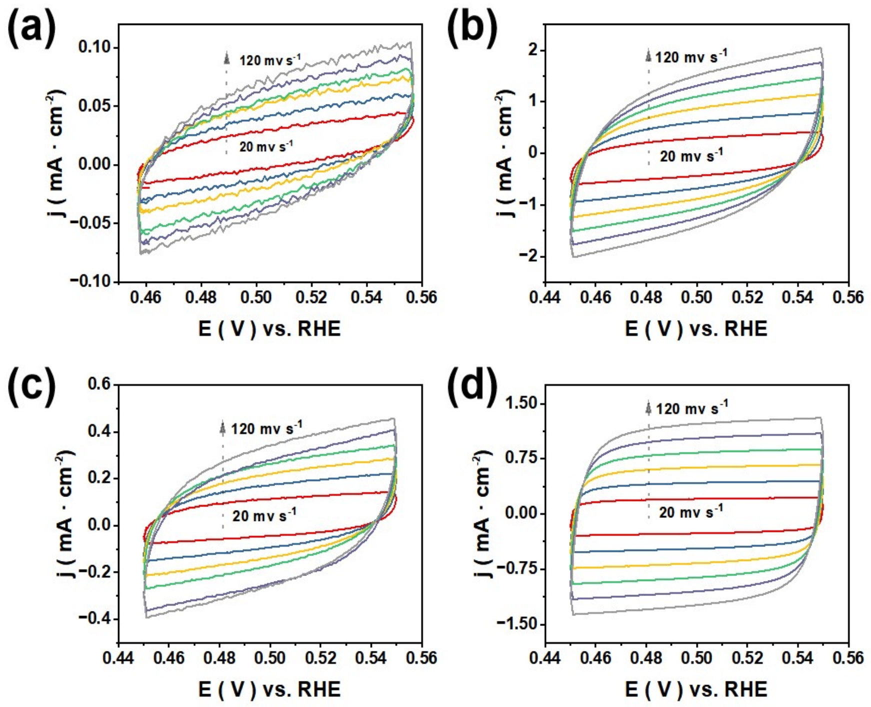

Figure S5 CV curves of (a) $Ni_2P-Ni_{12}P_5$, (b) $Os/Ni_2P-Ni_{12}P_5$, (c) Os/C and (d) Pt/C in 1.0 M KOH.

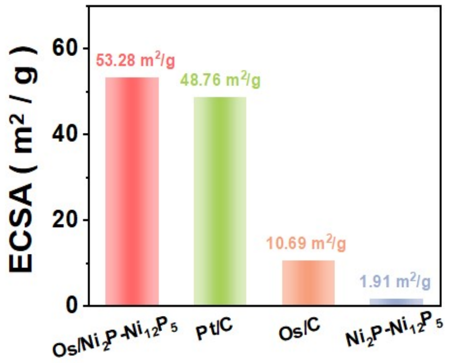

Figure S6 ECSA of different catalysts in 1.0 M KOH.

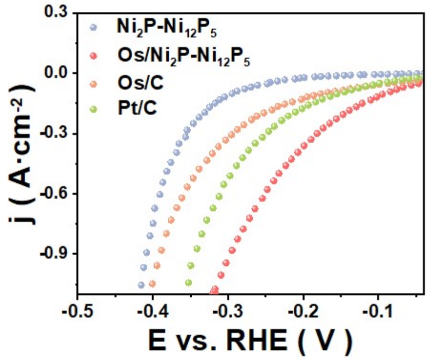

Figure S7 LSV curves at higher current density in 1.0 M KOH.

Figure S8 Stability test of $Os/Ni_2P\text{-}Ni_{12}P_5$ under -1.05 V in 1M KOH.

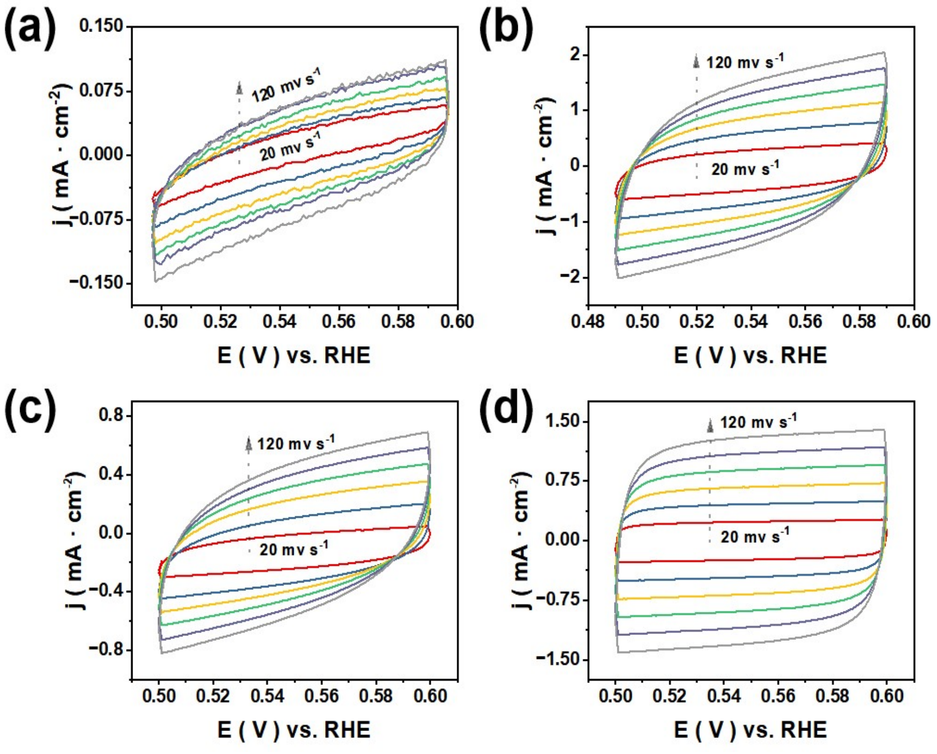

Figure S9 CV curves of (a) $Ni_2P\text{-}Ni_{12}P_5$, (b) $Os/Ni_2P\text{-}Ni_{12}P_5$, (c) Os/C and (d) Pt/C in 1.0 M KOH + seawater.

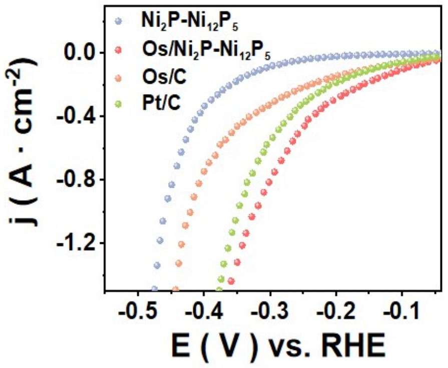

Figure S10 LSV curves at higher current density in 1.0 M KOH + seawater.

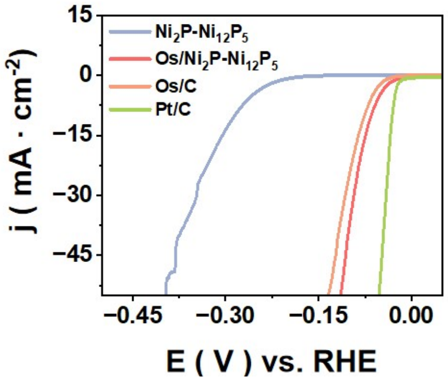

Figure S11 LSV curves of different catalysts in 0.5 M H_2SO_4.

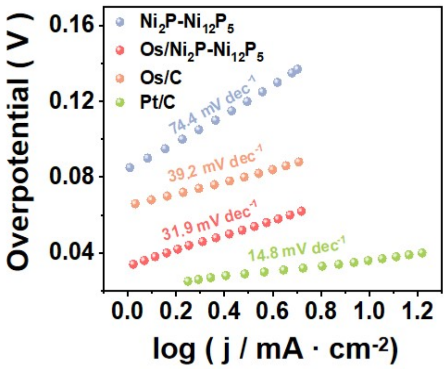

Figure S12 Tafel slopes of different catalysts in 0.5 M H_2SO_4.

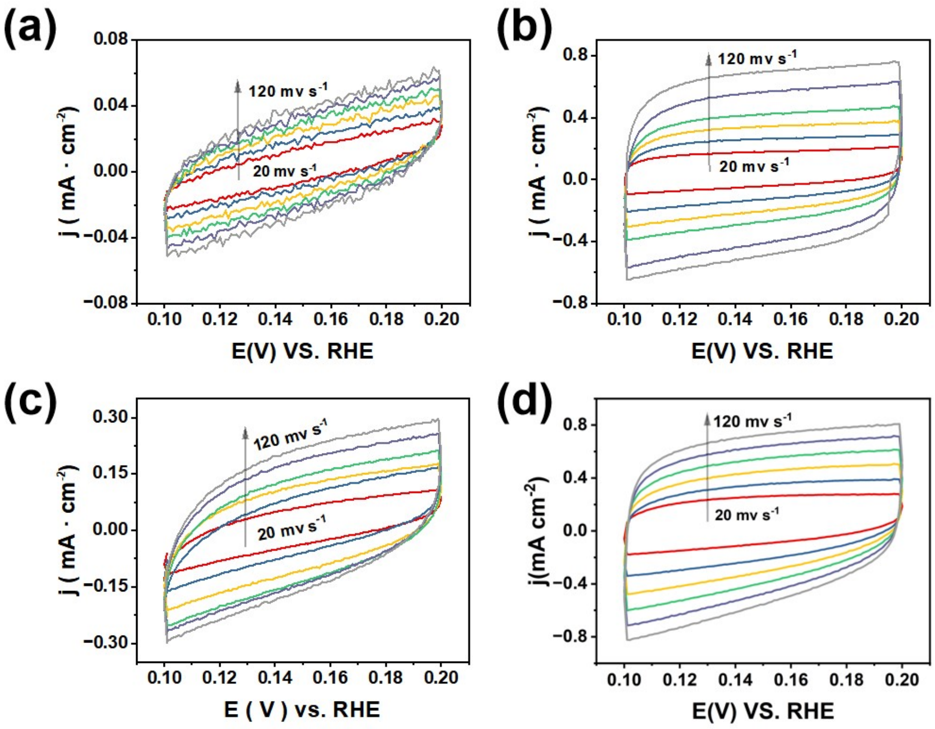

Figure S13 CV curves of (a) Ni_2P-$Ni_{12}P_5$, (b) Os/Ni_2P-$Ni_{12}P_5$, (c) Os/C and (d) Pt/C in 0.5 M H_2SO_4.

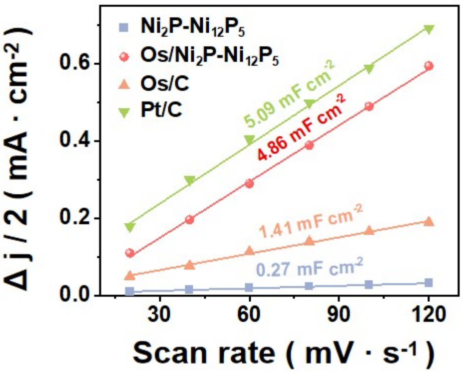

Figure S14 C_{dl} of different catalysts in 0.5 M H_2SO_4.

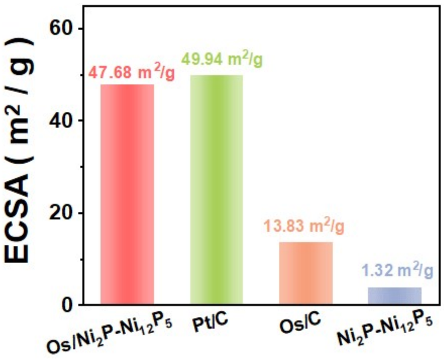

Figure S15 ECSA of different catalysts in 0.5 M H_2SO_4.

Figure S16 Nyquist plots of different catalysts in 0.5 M H_2SO_4.

Figure S17 LSV curves at higher current density in 0.5 M H_2SO_4.

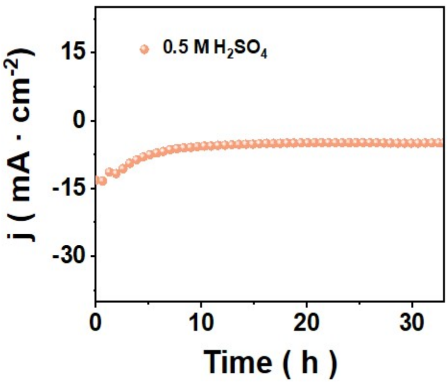

Figure S18 Stability test of Os/Ni_2P-$Ni_{12}P_5$ under -0.28 V in 0.5 M H_2SO_4.

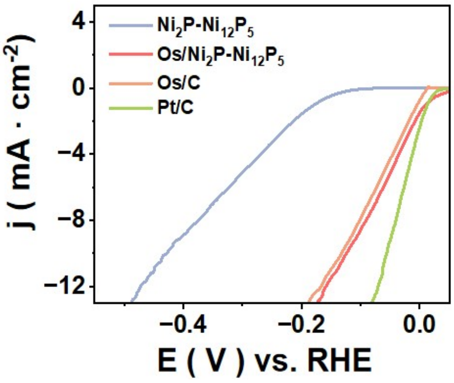

Figure S19 LSV curves of different catalysts in 1.0 M PBS.

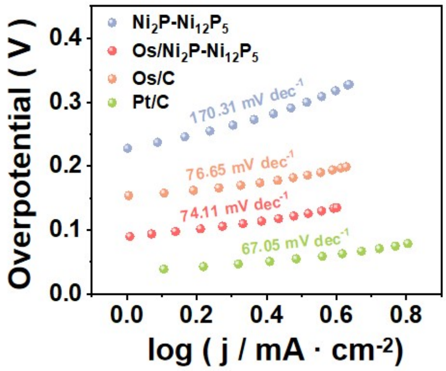

Figure S20 Tafel slopes of different catalysts in 1.0 M PBS.

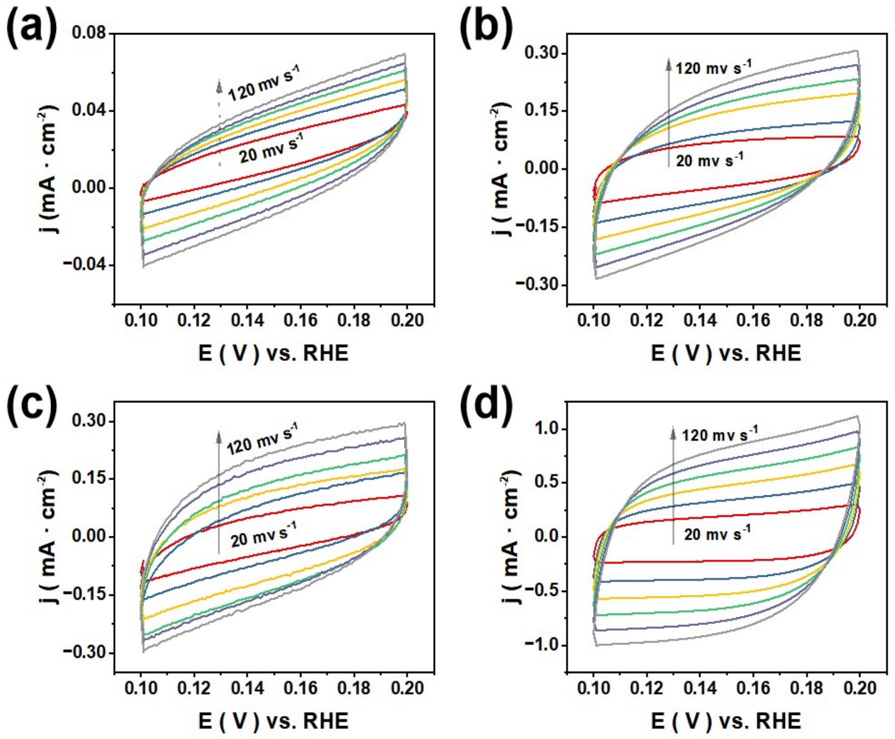

Figure S21 CV curves of (a) Ni_2P-$Ni_{12}P_5$, (b) Os/Ni_2P-$Ni_{12}P_5$, (c) Os/C and (d) Pt/C in 1.0 M PBS.

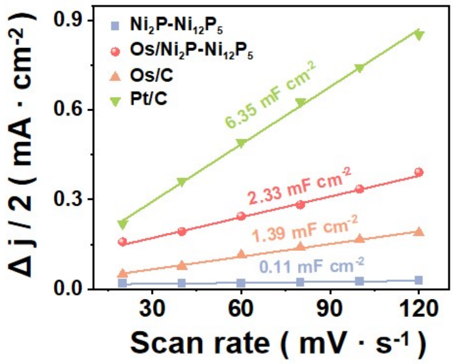

Figure S22 C_{dl} of different catalysts in 1.0 M PBS.

Figure S23 Nyquist plots of different catalysts in 1.0 M PBS.

Figure S24 LSV curves at higher current density in 1.0 M PBS.

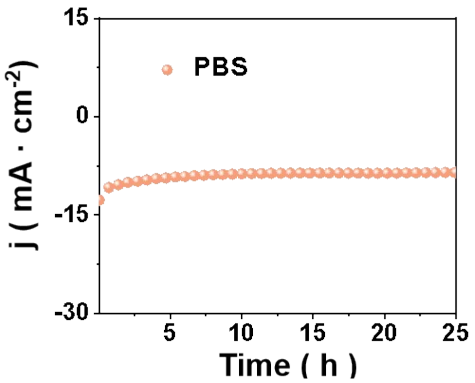

Figure S25 Stability test of Os/Ni_2P-$Ni_{12}P_5$ under -0.75 V in 1.0 M PBS.

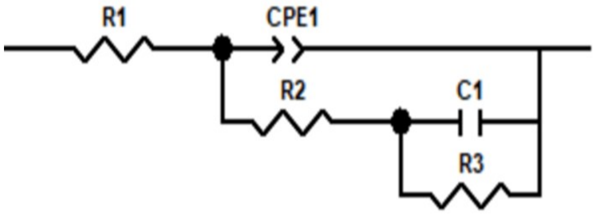

Figure S26 An equivalent circuit model made to simulate the Nyquist plots.

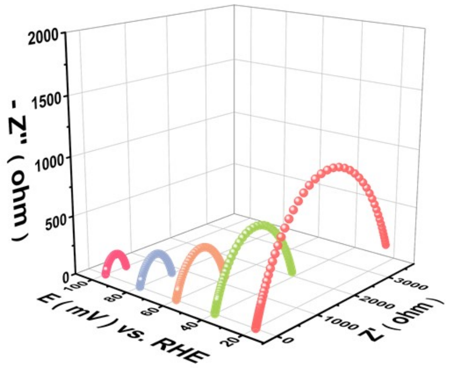

Figure S27 In-situ EIS of Ni_2P-$Ni_{12}P_5$ in 1.0 M KOH.

Figure S28 Bode Plots of Ni_2P-$Ni_{12}P_5$.

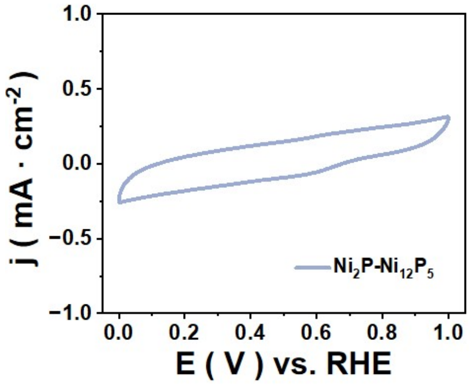

Figure S29 CV curve of Ni_2P-$Ni_{12}P_5$ at a scan rate of 50 mV s^{-1}.

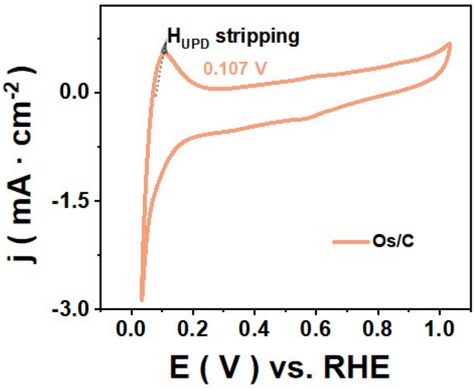

Figure S30 CV curve of Os/C at a scan rate of 50 mV s^{-1}.

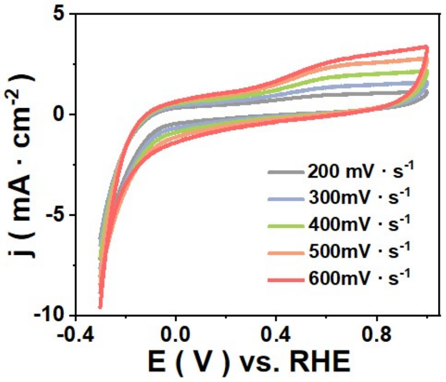

Figure S31 CV curves recorded at various scan rates of Ni_2P-$Ni_{12}P_5$ under Ar atmosphere.

Figure S32 CV curves recorded at various scan rates of Os/C under Ar atmosphere.

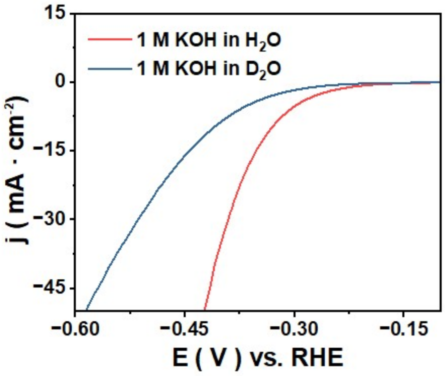

Figure S33 LSV curves of Ni_2P-$Ni_{12}P_5$ measured in 1.0 M KOH H_2O solution and 1.0 M KOH D_2O solution.

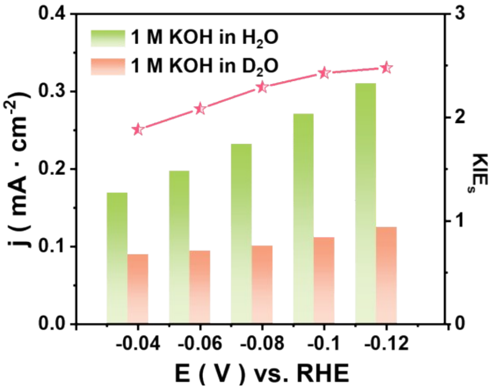

Figure S34 Calculated KIE values under corresponding potentials of Ni_2P-$Ni_{12}P_5$.

Figure S35 LSV curves of Os/C measured in 1.0 M KOH H_2O solution and 1.0 M KOH D_2O solution.

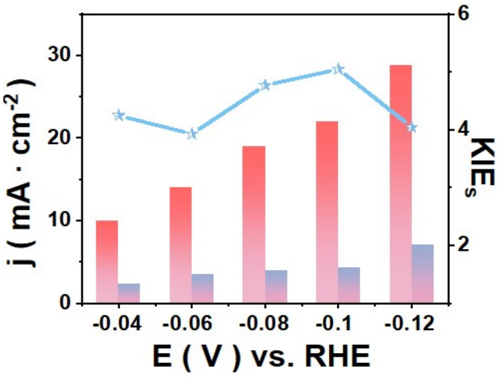

Figure S36 Calculated KIE values under corresponding potentials of Os/C.

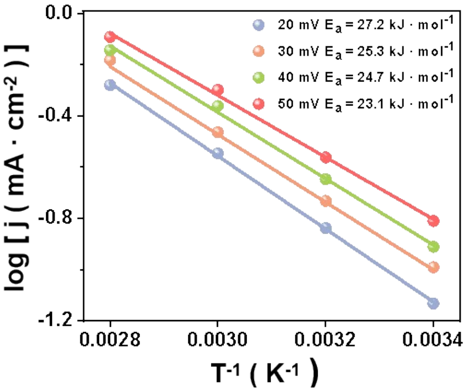

Figure S37 Arrhenius plots of Ni_2P-$Ni_{12}P_5$.

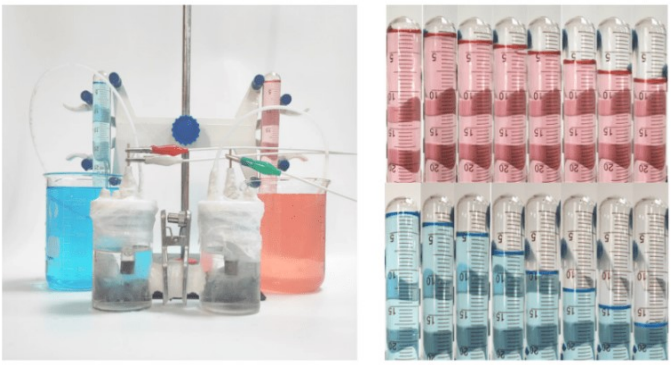

Figure S38 Photograph of the homemade two-electrode system used to measure Faradaic efficiency and photographs of gases produced at the cathode and anode change over time.

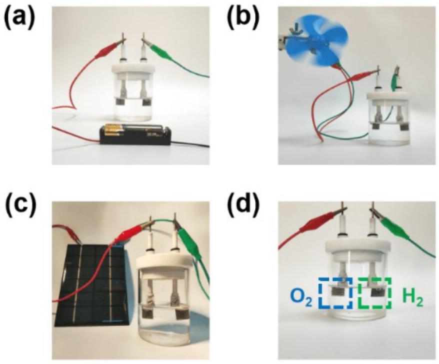

Figure S39 (a-c) Simulation of electricity, wind, and solar energy for H_2 generation and (d) O_2 and H_2 bubbles generated on the electrode surface.

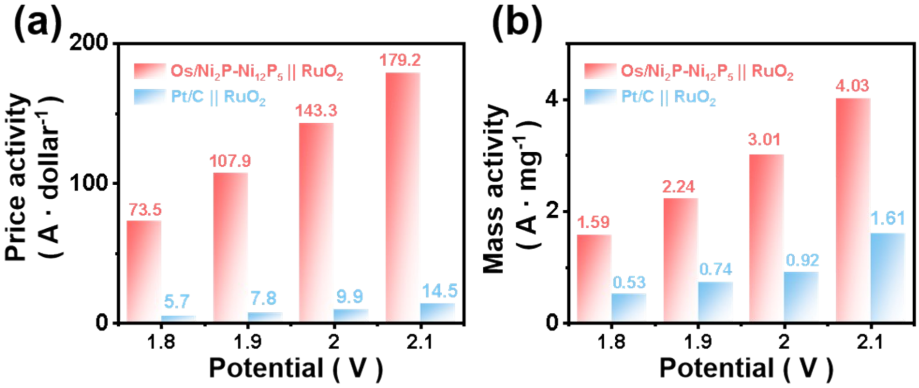

Figure S40 (a) price activities and (b) mass activities between Os/Ni_2P-$Ni_{12}P_5$ ‖ RuO_2 and Pt/C ‖ RuO_2 at various cell potentials.[5]

Table 1

The ICP result of Os in Os/Ni_2P-$Ni_{12}P_5$.

ICP	Os
wt %	9.4208%

Table 2

HER activity of various metal phosphide electrocatalysts in 1.0 M KOH.

Catalysts	Overpotential (mV)@10mA cm^{-2}	Tafel slope (mV dec^{-1})	Ref.
**Ag-AgP_2/Ni_2P**	78	68.9	6
**NiO@NiP/NF**	76	98	7
**Ni_5P_4/NF**	106	79	8
**$Fe_{0.074}$NiP/NWM**	108	52.1	9
**A-NiFeP/NiP-Fe:10%**	69.5	57.6	10
**Ni_5P_4**	110	105	11
**NiCoP/CC**	95	88.6	12
**Mo-NiP**	62	93.3	13
**Ni_2P/MnP_4/CF**	69	107	14
**Ni_2P/CF**	95	173	14
**Ru/Ni_2P@NPC**	132	124	15
**Ru doped $Ni(OH)_2$/TM-0.2**	135	63.7	16
**H-B/Ru-FeP**	110	76.7	17
**Os/Ni_2P-$Ni_{12}P_5$**	19	25.4	**This work**

Table 3

HER activity of various metal phosphide electrocatalysts in 1.0 M KOH + seawater.

Catalysts	Overpotential (mV) @100mA cm^{-2}	Tafel slope (mV dec^{-1})	Ref.
**V-$Ni_2P/Ni_{12}P_5$/NF**	215	109	18
**$Ni_2P/Ni_{12}P_5$/NF**	246	143	18
**Co-NiP@VP/NF**	164	65.41	19
**Cr-NiP@VP/NF**	184	94.07	19
**Ni_2P-Fe_2P/NF**	252	86	20
**$NiMoO_4$@NiFeP**	188	89.3	21
**Mo-NiP@NF**	102	57.4	22
**A-NiFeP/NiP-Fe:10%**	127.6	60.3	6
**Ru-CoP_2**	64	111.3	23
**Os/Ni_2P-$Ni_{12}P_5$**	80.7	45.6	**This work**

Table 4

HER activity of various metal phosphide electrocatalysts in 0.5 M H_2SO_4.

Catalysts	Overpotential (mV) @10mA cm^{-2}	Tafel slope (mV dec^{-1})	Ref.
**NiP/CC**	225	96.7	12
**NiCoP/CC**	197	86.7	12
**FeP**	66	55	24
**Co_2P/NPPC-800**	121	71	25
**Ni_2P/Ti**	52	68.4	26
**Ni_2P@B, N-GC**	89	69.3	27
**Fe_2P-Ni_2P/Co_2P**	89.7	48	28

**$Os/Ni_2P\text{-}Ni_{12}P_5$**	65	31.9	**This work**

Table 5

HER activity of various metal phosphide electrocatalysts in 1.0 M PBS.

Catalysts	Overpotential (mV) @10mA cm^{-2}	Tafel slope (mV dec^{-1})	Ref.
**Ni_2P@B, N-GC**	113	104.6	27
**$Fe_2P\text{-}Ni_2P/Co_2P$**	212	135	28
**Ni_2P/Ti**	109	106.8	26
**NiP_2 NSs/CC**	136	103	29
**$NiPS_3$**	212	115	30
**$Os/Ni_2P\text{-}Ni_{12}P_5$**	136	74.11	**This work**

Table 6

The comparison of AEM water electrolyser using transition metal-based electrocatalysts in alkaline media at a current density of 500 mA cm^{-2}

AEMWE	Electrolyte	E_{cell}/V	Stability	Ref.
**Pt/WN@CP || NiFe-LDH@NF**	25°C 1 M KOH + seawater	1.90 V	500 mA cm^{-2}@120 h	31
**Ni/WN@CP-90 || NiFe-LDH@NF**	25°C 1 M KOH + seawater	1.97 V	500 mA cm^{-2}@120 h	31
**P-Pt/$NiMoO_4$@NF || S-(Ni,Fe)OOH**	60°C 1 M KOH + seawater	1.75 V	500 mA cm^{-2}@120 h	32
**Ru-Ni_2P/Fe_2P || Ru-Ni_2P/Fe_2P**	60°C 1 M KOH + seawater	1.81 V	500 mA cm^{-2}@100 h	33
**$NiCoP_v$@NF || $NiCoP_v$@NF**	60°C 1 M KOH + seawater	2.43 V	500 mA cm^{-2}@110 h	34

**Os/Ni_2P-$Ni_{12}P_5$ || RuO_2**	60℃ 1 M KOH + seawater	1.84 V	500 mA cm^{-2}@80 h	**This work**

Table 7

Prices of six platinum group metals.

Metal	Symbol	Unit of measure	U.S.
**Platinum**	Pt	troy ounce	1090
**Iridium**	Ir	troy ounce	4600
**Ruthenium**	Ru	troy ounce	465
**Palladium**	Pd	troy ounce	1560
**Rhodium**	Rh	troy ounce	7550
**Osmium**	Os	troy ounce	**390**

The prices for various platinum group metals are from the BASF corporation website on Feb. 2, 2025. (https://chemical-catalysts-and-adsorbents.basf.com/global/en)

Reference

1 Q. Li, X. Fu, H. Li, Z. Xiao, G. Xu, D. Chen, C. Li, W. Jin, T. Ma, Z. Wu, L. Wang and S. Feng, *Adv. Funct. Mater.*, 2024, **34**, 2408517

2 X. Liu, X. Wang, K. Li, J. Tang, J. Zhu, J. Chi, J. Lai and L. Wang, *Angew. Chem. Int. Ed.*, 2024, **63**, e202316319

3 P. Yang, F. Liu, X. Zang, L. Xin, W. Xiao, G. Xu, H. Li, Z. Li, T. Ma, J. Wang, Z. Wu and L. Wang, *Adv. Energy Mater.*, 2024, **14**, 2303384

4 X. Kang, F. Yang, Z. Zhang, H. Liu, S. Ge, S. Hu, S. Li, Y. Luo, Q. Yu, Z. Liu, Q. Wang, W. Ren, C. Sun, H.-M. Cheng and B. Liu, *Nat. Commun.*, 2023, **14**, 3607

5 Y. Zhu, M. Klingenhof, C. Gao, T. Koketsu, G. Weiser, Y. Pi, S. Liu, L. Sui, J. Hou, J. Li, H. Jiang, L. Xu, W.-H. Huang, C.-W. Pao, M. Yang, Z. Hu, P. Strasser and J. Ma, *Nat. Commun.*, 2024, **15**, 1447

6 J. Wang, F. Tian, L. Zhang, H. Zhang, J. Fan, L. Zhang, T. Xu and X. Cui, *J. Colloid Interface Sci.*, 2024, **673**, 284-290

7 C. Sun, H. Wang, J. Ren, X. Wang and R. Wang, *Nanoscale.*, 2021, **13**, 13703-13708

8 Z. Qiu, Y. Dai, F. Yang, R. Zhang, W. Guo, X. Xiao, Y. Tong, L. Yao and Z. Yang, *J. Alloys Compd.*, 2024, **976**, 173029

9 B. Hui, J. Li, Y. Lu, K. Zhang, H. Chen, D. Yang, L. Cai and Z. Huang, *J. Energy Chem.*, 2021, **56**, 23-33

10 S.-Y. Lu, L. Wang, C. Wu, J. Zhang, W. Dou, T. Hu, R. Wang, Y. Liu, Q. Yang and M. Jin, *Inorg. Chem. Front*., 2024, **11**, 3187-3199

11 N. Mushtaq, Y. Bai, Alamgir, C. Cao, U. Khan and J. Gao, *Mater. Lett.*, 2024, **371**, 136920

12 K. Jiang, J. Li, Z. Zheng, T. Zhang, G. Wang, C. Shi and X. Hou, *ACS Appl. Energy Mater.*, 2024, **7**, 7895-7905

13 J. Nie, J. Shi, L. Li, M.-Y. Xie, Z.-Y. Ouyang, M.-H. Xian, G.-F. Huang, H. Wan, W. Hu and W.-Q. Huang, *Adv. Energy Mater.*, 2025, **15**, 2404246

14 R. Yan, X. Zou, Y. Liang, Y. Liu, F. Hu and Y. Mi, *J. Colloid Interface Sci.*, 2024, **669**, 349-357

15 J.-Q. Chi, X.-Y. Zhang, X. Ma, B. Dong, J.-Q. Zhang, B.-Y. Guo, M. Yang, L. Wang, Y.-M. Chai and C. Liu, *ACS Sustain. Chem. Eng.*, 2019, **7**, 17714-17722

16 Y. Wang, J. Wang, T. Xie, Q. Zhu, D. Zeng, R. Li, X. Zhang and S. Liu, *Appl. Surf. Sci.*, 2019, **485**, 506-512

17 Z. Wang, Y. Wang, W. Xiao, X. Wang, Y. Fu, G. Xu, Z. Li, Z. Wu and L. Wang, *J. Mater. Chem. A*., 2022, **10**, 15155-15160

18 D. He, L. Cao, J. Huang, X. Zhang, C. Wang, K. Li, K. Kajiyoshi and L. Feng, *Mol. Catal*., 2024, **558**, 114046

19 M. Liu, H. Zhao, X. Du and X. Zhang, *Fuel*., 2025, **381**, 133717

20 H. Tang, Y. Qi, D. Feng, Y. Chen, L. Liu, L. Hao, K. Yue, D. Li and Y. Wang, *Sustain. Energy & Fuels*., 2022, **6**, 4477-4483

21 Y. Cong, K. Chen, X. Chen, W. Xu, A. Cai and T.-T. Li, *Inorg. Chem*., 2023, **62**, 4960-4970

22 W. Hao, X. Ma, L. Wang, Y. Guo, Q. Bi, J. Fan, H. Li and G. Li, *Adv. Energy Mater.*, 2025, **15**, 2403009

23 B. Deng, J. Shen, J. Lu, C. Huang, Z. Chen, F. Peng and Y. Liu, *J. Energy Chem*., 2025, **100**, 317-326

24 A. Zarebidaki and M. Alimagham, *Fuel*., 2025, **392**, 134898

25 S. Jia, Y. Cheng, Q. Huang, Q. Li, Q. Zhang, Z. Wang, Y. Zhang, N. Zhang and Y. Mu, *Int. J. Hydrogen Energy*., 2022, **47**, 24796-24806

26 Y. Yan, Y. Chen, M. Shao, J. Hou, X. Chen, L. Fan, F. Kong and M. Chen, *Fuel*., 2024, **367**, 131551

27 C. Lyu, J. Cheng, Y. Yang, W.-M. Lau, N. Wang, Q. Wu and J. Zheng, *J. Colloid Interface Sci.*, 2023, **651**, 93-105

28 W. Chen, S. Chen, M. Guo, X. Jiang, J. Xia, M. Chen, Y. Xiong and X. Qian, *Int. J. Hydrogen Energy*., 2024, **78**, 851-860

29 X. Wang, H. Zhou, D. Zhang, M. Pi, J. Feng and S. Chen, *J. Power Sources*., 2018, **387**, 1-8

30 J. Zhang, R. Cui, X. a. Li, X. Liu and W. Huang, *J. Mater. Chem. A*., 2017, **5**, 23536-23542

31 S. Geng, L. Chen, Y. Wu, Y. Wang and S. Song, *J. Energy Chem*., 2025, **105**, 302-311

32 S. Xu, J. Chi, T. Cui, Z. Li, F. Liu, J. Lai and L. Wang, *Nano Energy*., 2024, **126**, 109698

33 X. Li, T. Wu, N. Li, S. Zhang, W. Chang, J. Chi, X. Liu and L. Wang, *Adv. Funct. Mater.*, 2024, **34**, 2400734

34 L. Guo, J. Chi, T. Cui, J. Zhu, Y. Xia, H. Guo, J. Lai and L. Wang, *Adv. Energy Mater.*, 2024, **14**, 2400975
